# Biological Effects of Maslinic Acid on Human Epithelial Cells Used in Tissue Engineering

**DOI:** 10.3389/fbioe.2022.876734

**Published:** 2022-04-27

**Authors:** Olimpia Ortiz-Arrabal, Jesús Chato-Astrain, Pascual Vicente Crespo, Ingrid Garzón, María Dolores Mesa-García, Miguel Alaminos, Carolina Gómez-Llorente

**Affiliations:** ^1^ Tissue Engineering Group, Department of Histology, Faculty of Medicine, University of Granada, Granada, Spain; ^2^ Instituto de Investigación Biosanitaria Ibs.GRANADA, Granada, Spain; ^3^ Doctoral Program in Biochemistry and Molecular Biology, University of Granada, Granada, Spain; ^4^ Department of Biochemistry and Molecular Biology II, School of Pharmacy, Campus Universitario de Cartuja, Granada, Spain; ^5^ Biomedical Research Center, Institute of Nutrition and Food Technology “José Mataix”, University of Granada, Granada, Spain

**Keywords:** maslinic acid, tissue engineering, epithelial cells, cell culture, cell proliferation

## Abstract

In the present work, we evaluated the potential of maslinic acid (MA) to improve currently available keratinocyte culture methods for use in skin tissue engineering. Results showed that MA can increase cell proliferation and WST-1 activity of human keratinocytes after 24, 48, and 72 h, especially at the concentration of 5 μg/ml, without affecting cell viability. This effect was associated to a significant increase of KI-67 protein expression and upregulation of several genes associated to cell proliferation (PCNA) and differentiation (cytokeratins, intercellular junctions and basement membrane related genes). When human keratinocytes were isolated from skin biopsies, we found that MA at the concentration of 5 μg/ml significantly increased the efficiency of the explant and the cell dissociation methods. These results revealed the positive effects of MA to optimize human keratinocyte culture protocols for use in skin tissue engineering.

## 1 Introduction

The human skin is the largest organ in the body and it plays an important role as the external protective barrier against microorganisms, chemicals and mechanical forces, and is able to prevent from water loss and regulate body temperature (Proksch et al., 2008). Most of these functions are carried out by the epidermis, the most external layer of the skin consisting of a stratified multilayered epithelium with different degrees of keratinization (Proksch et al., 2008; Lefèvre-Utile et al., 2021). Severe skin damage, especially large and deep burns, is a life-threatening condition and a therapeutic challenge. In general, the gold-standard treatment of burnt patients is the use of autologous skin grafts obtained from nonaffected areas of the patient skin (Böttcher-Haberzeth et al., 2010). However, the lack of donor sites in patients affected by large burns (Shevchenko et al., 2010) and the morbidity associated to this treatment make necessary the search for alternative therapeutic strategies (Orgill and Ogawa, 2013).

Therefore, several models of bioartificial human skin have been developed in the last years by tissue engineering (Gómez et al., 2011; Carriel et al., 2012; Holmes et al., 2019; Momeni et al., 2019). Three main components are combined in all these models: living cells, biocompatible biomaterials and growth factors (Tada et al., 2012). One of the human skin substitutes currently used in the clinical management of severely burnt patients is the UGRSKIN model consisting of a nanostructured fibrin-agarose hydrogel containing dermal fibroblasts with a superficial layer of epidermal keratinocytes (Carriel et al., 2012). Despite its potential as an advanced therapies medicinal product (ATMP) for the treatment of severely burnt patients (Egea-Guerrero et al., 2019), including adult and pediatric patients, UGRSKIN requires the establishment of keratinocyte cultures from a skin biopsy obtained from the patient. One of the main limitations of this ATMP is the long time required to generate abundant cell cultures, since most human epithelial cells, including skin keratinocytes, are slow-cycling cells that show low proliferation rates (Wang et al., 2021). The clinical need to rapidly cover the damaged areas of the skin patient makes necessary to develop new cell culture protocols able to shorten the biofabrication time of this ATMP (Gardien et al., 2014; Chato-Astrain et al., 2021). In general, most protocols used for the establishment of human skin keratinocyte primary cell cultures are the cell dissociation and the explant techniques (Breidahl et al., 1989; Chato-Astrain et al., 2021). In the first case, epidermal cells are dissociated by enzymatically disrupting the intercellular junctions using trypsin or other enzymes to generate a keratinocyte cell suspension that can be furtherly cultured in culture flasks (Llames et al., 2004; Dearman et al., 2021). In the second case, small tissue biopsies are placed on the surface of a culture flask where cells directly migrate and expand from the explanted tissue to the culture surface (Bayar and Gulses, 2013; Shwetha et al., 2019); indeed, some reports suggest that this method could be more efficient than the cell dissociation technique (Kedjarune et al., 2001; Chato-Astrain et al., 2021). In both cases, novel biofabrication protocols are necessary to improve the efficiency of the procedure.

One of the potentially useful tools to improve cell proliferation and to optimize the biofabrication process is the use of culture media enriched with bioactive factors. Among others, previous reports demonstrated the partial usefulness of several factors such as the epithelial growth factor (EGF) free (Shirakata, 2010) or linked to nanostructured lipid carriers (NLC) (Chato-Astrain et al., 2021), vitamin D (Sakai and Demay, 2000) and histamine (Glatzer et al., 2013). However, the potential of most of these components is very limited.

Several natural molecules were recently proposed to exert a positive biological effect on human cell physiology. Although their role on human keratinocyte cultures has not been determined to date, it has been suggested that several components of the *Olea europaea* olive oil may be potentially beneficious for the skin due to their topical antioxidant effect (Nunes et al., 2021). In fact, it has been demonstrated that olive oil extracts favor skin wound healing through NOS-2 and NFE2L2factors (Schanuel et al., 2019, 2). One of the relevant components present in the olive oil extract is maslinic acid (MA), a pentacyclic triterpene present in skins and seeds of olive fruits, that is extracted in the pomace oil (Mokhtari et al., 2015). MA has demonstrated health benefits derived from its antioxidant, anti-inflammatory, antimicrobial, hepato-protective and anticancer properties (Sánchez-Quesada et al., 2013; Lozano-Mena et al., 2014; Jing et al., 2021), and this molecule could influence cell physiology in a dose-dependent manner (Yu et al., 2021). However, to our best knowledge, it potential effect for the stimulation of human skin keratinocyte cell cultures growth has not been described to the date. In the present study, we have evaluated the effects of MA on human skin keratinocyte cultures to determine its putative usefulness in tissue engineering protocols.

## 2 Materials and Methods

### 2.1 Immortalized Human Skin Keratinocyte Cell Cultures and Culture Conditions

In the first place, we evaluated the effects of MA on immortalized human skin keratinocytes using the commercially available cell line CRL-4048 from the American Type Culture Collection (ATCC). These cells were cultured in keratinocyte culture medium (KC medium) consisting of a 3:1 mixture of Dulbecco’s Modified Eagle Medium (DMEM) and Nutrient Mixture F-12 Ham supplemented with 10% fetal bovine serum (FBS), 1% antibiotics/antimycotics, 24 μg/ml adenine, 0.4 μg/ml hydrocortisone, 5 μg/ml insulin, 10 ng/μL epidermal growth factor and 1.3 ng/ml triiodothyronine (all these products, from Merck, Burlington, MA), using standard cell culture conditions (37°C and 5% CO_2_). The culture medium was renewed twice a week and cells were dissociated with CTS TrypLE Select Enzyme solution (Invitrogen, Waltham, MA) before reaching confluence.

To evaluate the effects of MA, we first carried out a wide-range analysis of increasing concentrations of MA (Biomaslinic S.L, Granada, Spain) using immortalized keratinocytes. In short, cells were subcultured in 96-well plates at a density of 7.25×10^3^ cells per well and allowed to attach for 24 h in KC medium. This low cell density allowed us to obtain non-confluent cell cultures at all study times. Then, the culture medium was replaced by KC medium containing different concentrations of MA (1, 5, 10, 20, 40, and 80 μg/ml) and cells were cultured in this medium for 24, 48 and 72 h. To prepare the different media containing increasing concentrations of MA, we first generated a 10 mg/mL MA stock solution by dissolving MA powder in dimethyl sulfoxide (DMSO) as recommended by the manufacturer. Then, this stock solution was sequentially diluted in KC to the desired final concentrations of MA. Cells cultured in KC medium without MA were used as controls (CTR), whereas cells treated with 2% triton X-100 (Probus, Barcelona, Spain) were used as negative controls of cell viability (NEG). The same concentration of DMSO solvent was used in all study groups, including the CTR. All these experiments were carried out using sextuplicates for each condition (n = 6 samples per study group).

### 2.2 Analysis of Cell Viability of Immortalized Keratinocytes Cultured in Increasing Concentrations of MA

First, the cell viability was assessed by quantifying the DNA released from the cells to the culture medium at each time and for each MA concentration. For this purpose, aliquots were taken from each well and the amount of DNA present in each sample was measured by determining the 260/280 nm absorbance using a NanoDrop 2000 spectrophotometer (Thermo Fisher Scientific, Waltham, MA). Results were normalized regarding the results obtained in CTR cells (considered as 100% viability).

Then, we used the LIVE/DEAD Viability/Cytotoxicity kit (Invitrogen) to evaluate cell viability of immortalized cells cultured in the different MA concentrations and incubation times. In brief, cells were washed twice in phosphate-buffered saline (PBS) and cultured in the LIVE/DEAD reagent prepared as recommended by the manufacturer. Then, cells were incubated for 5 min at room temperature in the dark. Finally, images were obtained using a ZOE Fluorescent Cell Imager (Biorad), and the numbers of green cells (live cells) and red cells (dead cells) were assessed in each sample.

### 2.3 Analysis of Cell Proliferation of Immortalized Keratinocytes Cultured in Increasing Concentrations of MA

The effect of MA on cell proliferation was first analyzed by quantifying the number of cells in each culture condition after each incubation period. Cells were washed twice in PBS and detached using CTS TrypLE Select Enzyme solution (Invitrogen) for 5 min. The detachment solution was then inactivated with 10% FBS, and the number of cells present in the solution was determined by flow cytometry using a NovoCyte flow cytometer (Acea Biosciences/Agilent Technologies, Santa Clara, CA).

Then, proliferation was evaluated by using the Cell Proliferation Reagent WST-1 (Roche, Basel, Switzerland). Cells were washed twice in PBS and the WST-1 reagent was added to each well diluted in culture medium, as recommended by the manufacturer. After 4 h of incubation at 37°C, the absorbance of the formazan dye -which correlates with the number of metabolically active, proliferating cells-was measured with an Asys UVM-340 scanning microplate reader (Biochrom/Harvard Bioscience, Holliston, MA).

### 2.4 Immunohistochemistry and Gene Expression Analysis of Immortalized Keratinocytes Cultured in 5 μg/ml of MA

Analysis of the results obtained in the previous experiment using a wide range of MA concentrations allowed us to select the concentration of 5 μg/ml of MA for the rest of the experiments carried out in the present work. By using the specific concentration of 5 μg/ml of MA, we first analyzed the percentage of cells showing positive expression of the cell proliferation marker KI-67 by immunohistochemistry. In brief, immortalized human keratinocytes were cultured in Lab-Tek II Chamber Slides (Thermo Fisher Scientific) at a density of 45×10^3^ cells per chamber, and KC medium or 5 μg/ml of MA medium was added for the CTR and study groups, respectively. After 24, 48 and 72 h, cells were fixed in 70% ethanol, washed in PBS and incubated with H_2_O_2_ to inactivate endogenous peroxidase. Non-specific sites were then blocked with 1x animal-free blocker and 1x casein was used for 30 min (both from Vector Laboratories, Burlingame, CA). Samples were then incubated overnight at 4°C with pre-diluted primary antibody against KI-67 (Vitro/Master Diagnostica, Granada, Spain), washed in PBS and incubated for 1 h with ready-to-use anti-rabbit secondary antibodies labelled with peroxidase. A diaminobenzidine substrate kit (Vitro/Master Diagnostica) was used to detect antibody binding and samples were then counterstained for 15 s with Harry’s Hematoxylin (Panreac/Applichem, Barcelona, Spain). Negative controls were processed following the same protocol, but the primary antibody against KI-67 was replaced by PBS. After staining, images were obtained using an Eclipse 90i Microscope (Nikon) and the percentage of KI-67-positive cells was quantified in each sample. In this case, eight samples were used for each condition (n = 8).

On the other hand, gene expression of cells cultured with KC and 5 μg/ml of MA media was assessed by real-time qRT-PCR. For this, immortalized human keratinocytes were cultured for 72 h in KC or 5 μg/ml of MA and total RNA was extracted and purified from each sample using a Qiagen RNeasy mini kit (Qiagen, Hilden, Germany). For the gene expression analysis, we designed a custom-made PRIME-PCR plate (Bio-Rad, Hercules, CA) containing 30 unique assays to evaluate one gene related to cell proliferation (*PCNA*), 9 cytokeratin genes (*KRT3, KRT5, KRT6A, KRT7, KRT8, KRT10, KRT13, KRT16* and *KRT19*), 9 genes related to intercellular junctions (*DSP, FN1, GJA1, GJA4, JUP, PKP1, PPL, TJP1,* and *TJP2*), 3 epithelial differentiation genes (*BGN, FLG,* and *IVL*) and 5 genes associated to the development of a basement membrane (*COL4A1, LAMA1, LAMA3, LAMB1,* and *LAMC1*), along with 3 control assays (*GAPDH*, RT and RQ). One µg of each RNA was retro-transcribed to cDNA using an iScript Advanced cDNA Synthesis Kit (Bio-Rad). Then, 1 µL of cDNA were mixed with 9 µL of H_2_O and 10 µL of SsoAdvanced Universal SYBR Green Supermix (Bio-Rad) and added to each well of the PRIME-PCR plate. A thermocycling protocol consisting of 40 amplification cycles with an annealing temperature of 60°C was then used with a Bio-Rad CFX Connect-96 instrument. Six samples were used for each condition (n = 6). Results were adjusted according to the efficiency of the reverse transcription reaction (RT control) and normalized to the *GAPDH* housekeeping gene expression using the CFX Manager 3.1 software provided by the manufacturer. Then, we calculated the ΔΔCq value for each gene, along with the relative gene expression (fold-change expression) of MA samples as compared to controls, and the status of each gene in MA samples (no changed, upregulated, or downregulated) using the CFX Manager 3.1 software.

### 2.5 Effects of MA on Primary Cell Cultures of Normal Human Skin Keratinocytes Established From Human Biopsies

To determine the potential utility of MA (5 μg/ml) to improve current keratinocyte isolation and cell culture protocols, we established primary cultures of normal human skin keratinocytes from skin biopsies using both the explant technique and the cell dissociation method. In both cases, foreskin was obtained from 6 pediatric patients (average age 6.8 years) subjected to circumcision. Tissues were washed in PBS containing 10% antibiotics and antimycotics (Merck), and 2 fragments with a diameter of 5 mm were obtained from each foreskin sample using a skin biopsy punch (Kai Medical, Seki, Japan). For the explant technique, each fragment was subsequently divided in four identical segments using a no. 10 surgical blade. Then, explants were placed in 6-well tissue culture-treated plates (Corning, Corning, NY) (4 explants per well and 2 wells per donor), with the epithelial part in contact with the culture surface to promote keratinocyte migration and proliferation on this surface. After 5 min, KC medium was carefully added to prevent explants from detaching. For the cell dissociation method, remaining tissues were digested in a cell dissociation solution containing 0.5 g/L of trypsin and 0.2 g/L of ethylenediaminetetraacetic acid (EDTA) (Merck) at 37°C for 30 min with slight agitation to detach the epithelial keratinocytes from the tissue. The supernatant was then harvested, mixed with 10% FBS to inactivate the trypsin and centrifuged at 1,000 rpm to obtain a pellet containing the detached cells. This pellet was then resuspended in a small volume of KC and the digestion process was repeated with the remaining tissue up to 3 times. Resuspended cells corresponding to the three digestion cycles were mixed and seeded in 6-well plates at a density of 10^5^ cells per well. In both cases (explant and cell dissociation methods), samples (12 wells containing 4 explants each) were cultured in KC medium (CTR) and samples (12 wells containing 4 explants each) were cultured in KC medium supplemented with 5 μg/ml of MA using exactly the same culture conditions. The culture medium was renewed twice a week.

For samples processed using the explant technique, images were obtained after 7, 14, and 21 days of follow-up using a ZOE Fluorescent Cell Imager (Biorad), and the percentage of explants succeeding in generating skin keratinocyte colonies was quantified for each condition (CTR or 5 μg/ml of MA). For the explant and cell dissociation samples, culture plates containing the cell cultures generated from each sample were fixed in formalin and stained with hematoxylin and eosin (Panreac/Applichem), and macroscopical images were taken. Then, the area corresponding to keratinocyte cells was quantified in each plate using the ImageJ 1.53e software (National Institutes of Health) as previously described (Vela-Romera et al., 2019). In brief, each macroscopical image corresponding to a specific well of the culture plate was converted to an 8-bit black and white binary image. Then, the total surface of the culture well and the surface specifically occupied by cultured cells were measured by the program by using the area fraction tool, and the percentage of area corresponding to cells was calculated for each well. Results were then expressed as the average and standard deviation of the 10 wells corresponding to each culture condition.

### 2.6 Statistical Analyses

For each type of analysis, results obtained in each experimental condition were compared with the control group. As none of the distributions fulfilled the criteria of normality using the Shapiro-Wilk test, non-parametric statistics was used for all comparisons. Comparison of the results of the LIVE/DEAD, WST-1, immunohistochemistry, cell number quantification and colony area analysis were carried out using Mann-Whitney tests. To compare the results obtained for the DNA quantification analysis expressed as percentage of the CTR group, and the percentage of explants able to successfully generate keratinocyte colonies, we used the Exact Test of Fisher. All these statistical tests were performed using the SPSS v25 software (IBM, Armonk, NY). *p* values below 0.05 were considered statistically significant, and all tests were carried out double-tailed.

## 3 Results

### 3.1 Cell Viability of Immortalized Human Keratinocytes Cultured With Increasing Concentrations of MA

**FIGURE 1:**
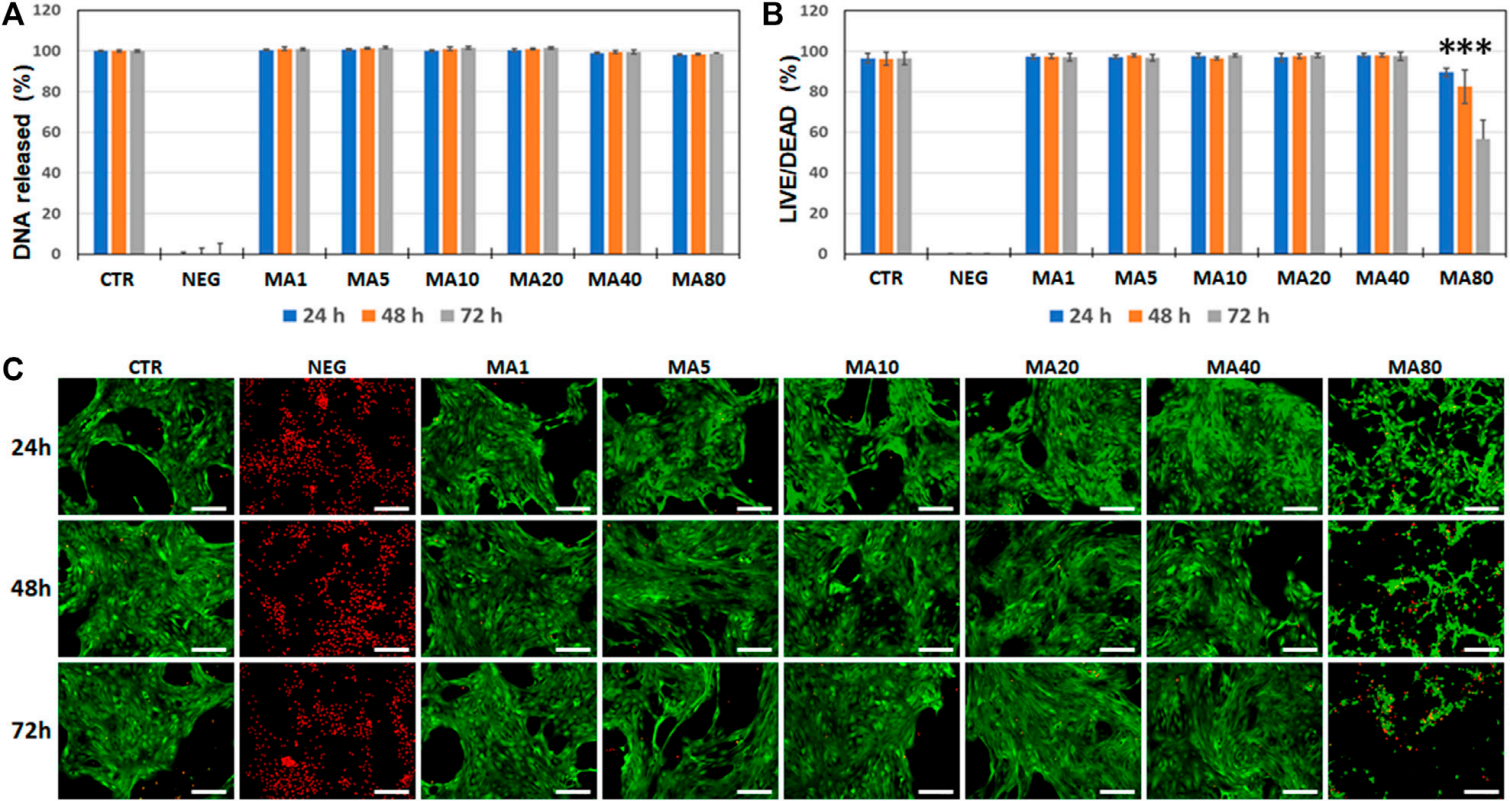
Analysis of cell viability of immortalized human keratinocytes cultured in different types of media for 24, 48 and 72 h. Cells were cultured in control culture medium (CTR) and media containing increasing concentrations of maslinic acid (MA1, MA5, MA10, MA20, MA40, and MA80, corresponding to 1, 5, 10, 20, 40, and 80 μg/ml of MA, respectively). As negative control (NEG), cells were incubated with 2% triton X-100. **(A)**: average values of cell viability as determined by quantification of DNA released to the medium after normalizing with respect to the CTR values. **(B)**: average values of cell viability as determined by LIVE/DEAD. In **(A,B)**, error bars correspond to standard deviations and asterisks show statistically-significant differences with CTR. **(C)**: fluorescence microscopy images of cells stained with the LIVE/DEAD system. Live cells are labeled in green and dead cells are labeled in red. Scale bars: 200 µm.

In the first place, we analyzed the potential cytotoxic effects of MA on an immortalized cell line of human keratinocytes by using a double approach. On one hand, we quantified the DNA released from dead cells to the culture medium. Results of the DNA quantification analysis showed that the cell viability was very high in all study groups (above 98% in all conditions). All groups were significantly higher than the NEG group (*p* < 0.001 for all comparisons), but no differences were found between the different MA concentrations and the CTR group (Figure 1A). On the other hand, we quantified the percentages of live and dead cells using the LIVE/DEAD method. Results of this analysis confirmed that MA did not significantly affect cell viability when the lowest concentrations (1–40 μg/ml) were used for 24 and 48 h. However, media containing 80 μg/ml of MA resulted in a significant reduction of cell viability at all study times compared to CTR (*p* = 0.0039). Results of the LIVE/DEAD analysis are shown in Figures 1B,C, and images corresponding to the green and red channels are shown in Supplementary Figure S1.

### 3.2 Pro-proliferative Effects of MA on Immortalized Human Keratinocytes

**FIGURE 2:**
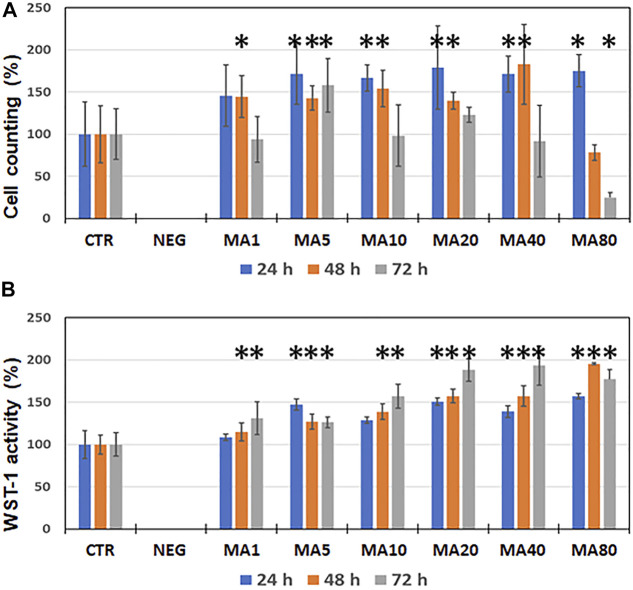
Analysis of cell proliferation of immortalized human keratinocytes cultured for 24, 48 and 72 h as determined by sequential cell number quantification (panel **(A)**) and WST-1 activity (panel **(B)**). Results are shown as average percentage values normalized to negative controls (NEG) and positive controls (CTR), which were considered as 0 and 100%, respectively. Error bars correspond to standard deviations. Statistically significant differences with CTR are labeled with asterisks (*).

In the second place, we analyzed the effects of increasing concentrations of MA on cell proliferation using immortalized human keratinocytes. In general, we found that specific concentrations of MA were associated to a significant increase of the cell number as compared to control cells, especially after 24 and 48 h of culture. Specifically, we found that cells cultured with 5 μg/ml of MA showed a significant cell number increase at the three time points analyzed (24, 48 and 72 h), whereas cells cultured with 1 μg/ml of MA showed higher number of cells than CTR only at 48 h. In turn, the presence of MA at 10, 20 and 40 μg/ml was associated to a significant increase at 24 and 48 h, whilst MA at 80 μg/ml showed a significant increase of the cell number at 24 h and a significant decrease at 72 h (Figure 2A and Supplementary Table S1).

In addition, we found that the cell proliferation activity as determined by WST-1 was also influenced by the presence of MA in the culture medium. As shown in Figure 2B and Supplementary Table S1, WST-1 activity was significantly increased in cells cultured for 24 h in media containing 5, 20, 40, and 80 μg/ml of MA, and in cells cultured in all concentrations of MA at 48 and 72 h.

**FIGURE 3:**
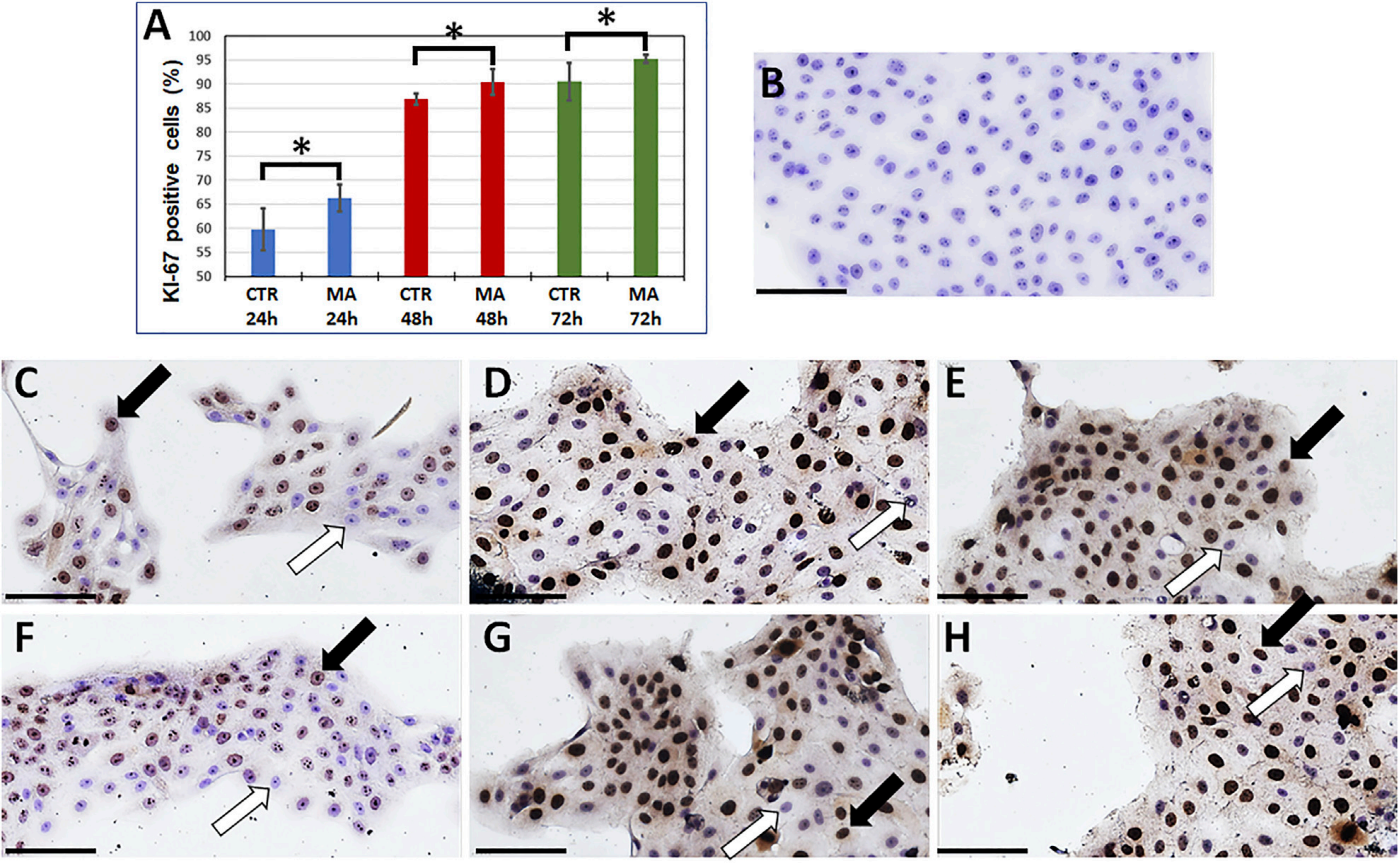
Immunohistochemical analysis of the cell-proliferation marker KI-67 in immortalized human keratinocytes cultured presence or absence of maslinic acid. The cells were cultured in the presence of 5 μg/ml of maslinic acid (MA) or in absence of this compound (Control CTR) for 24, 48 or 72 h. **(A)**: Percentage of cells showing KI-67 positive signal in each study group. Error bars represent standard deviations, and asterisks show statistically-significant differences with CTR for the Mann-Whitney test. **(B)**: Histological image of negative control cells (no primary antibody) showing no expression of KI-67. **(C–H)**: Illustrative images of cells showing positive and negative cells in the different study groups. **(C,D,E)**: CTR; **(F,G,H)**: MA at the concentration of 5 μg/ml; **(C,F)**: 24 h, **(D,G)**: 48 h, **(E,H)**: 72 h. Some cells showing positive expression of KI-67 are labeled with black arrows, and white arrows correspond to negative cells. Scale bars: 100 µm.

Once we determined that a concentration of 5 μg/ml of MA was able to improve cell proliferation on immortalized human keratinocytes at all study times, we performed specific immunohistochemical analyses using the cell proliferation marker KI-67. In this regard, our previous results were confirmed by the percentage of cells showing positive expression of this protein, that was significantly higher in the presence of 5 μg/ml of MA than in CTR at 24 h (*p* = 0.0087), 48 h (*p* = 0.0260) and 72 h (*p* = 0.0087) (Figure 3).

### 3.3 Gene Expression Analysis of Immortalized Human Keratinocytes Cultured With MA

Analysis of 27 genes determined by qRT-PCR showed that 5 genes were upregulated in the presence of MA at the concentration of 5 μg/ml (Table 1). First, we found that the cell-proliferation gene *PCNA* was upregulated in the MA group as compared to CTR, with more than 80% overexpression. Then, the analysis of several cytokeratin genes showed that most genes tended to increase in the presence of MA, although differences were significant only for *KRT3* being upregulated more than 300%. Similarly, most intercellular junction genes increased their expression in MA culture, and within them, *PPL* became upregulated more than 60%. The same trend was found for the epithelial differentiation and basement membrane-related genes, with *BGN* and *COL4A1* upregulated in the MA group (more than 60% in the case of *BGN* and more than 200% for *COL4A1*).

**TABLE 1:** Gene expression analysis of immortalized human keratinocytes cultured with maslinic acid. Cells cultures with 5 μg/mL MA as compared to cells cultured in control medium (CTR). For each gene, the relative fold-change expression of the cells cultured in 5 μg/ml of MA as compared to controls (CTR) is shown. The column at the right indicates whether the expression of each gene is different in the MA group as compared to CTR (upregulated, downregulated or no change).

Gene type	Gene symbol	Gene name	MA 5 μg/ml Vs. CTR FC	Expression
Proliferation	*PCNA*	Proliferating cell nuclear antigen	1.87	Up regulated (p = 0.028)
Cytokeratins	*KRT3*	Cytokeratin 3	3.03	Up regulated (p = 0.028)
	*KRT5*	Cytokeratin 5	1.40	No change (*p* > 0.05)
	*KRT6A*	Cytokeratin 6A	1.05	No change (*p* > 0.05)
	*KRT7*	Cytokeratin 7	1.03	No change (*p* > 0.05)
	*KRT8*	Cytokeratin 8	0.94	No change (*p* > 0.05)
	*KRT10*	Cytokeratin 10	1.03	No change (*p* > 0.05)
	*KRT13*	Cytokeratin 13	1.47	No change (*p* > 0.05)
	*KRT16*	Cytokeratin 16	1.06	No change (*p* > 0.05)
	*KRT19*	Cytokeratin 19	1.42	No change (*p* > 0.05)
Intercellular junctions	*DSP*	Desmoplakin	0.93	No change (*p* > 0.05)
	*FN1*	Fibronectin 1	0.84	No change (*p* > 0.05)
	*GJA1*	Gap Junction Protein Alpha 1	1.23	No change (*p* > 0.05)
	*GJA4*	Gap Junction Protein Alpha 4	0.97	No change (*p* > 0.05)
	*JUP*	Junction Plakoglobin	1.44	No change (*p* > 0.05)
	*PKP1*	Plakophilin 1	1.06	No change (*p* > 0.05)
	*PPL*	Periplakin	1.66	Up regulated (p = 0.028)
	*TJP1*	Tight Junction Protein 1	1.16	No change (*p* > 0.05)
	*TJP2*	Tight Junction Protein 2	1.04	No change (*p* > 0.05)
Epithelial differentiation	*BGN*	Biglycan	1.70	Up regulated (p = 0.028)
	*FLG*	Filaggrin	1.14	No change (*p* > 0.05)
	*IVL*	Involucrin	1.16	No change (*p* > 0.05)
Basement membrane	*COL4A1*	Collagen Type IV Alpha 1 Chain	2.05	Up regulated (p = 0.028)
	*LAMA1*	Laminin Subunit Alpha 1	0.85	No change (*p* > 0.05)
	*LAMA3*	Laminin Subunit Alpha 3	1.34	No change (*p* > 0.05)
	*LAMB1*	Laminin Subunit Beta 1	1.48	No change (*p* > 0.05)
	*LAMC1*	Laminin Subunit Gamma 1	1.16	No change (*p* > 0.05)

### 3.4 Establishment of Primary Keratinocyte Cultures From Human Skin Biopsies Using Media With MA

**FIGURE 4:**
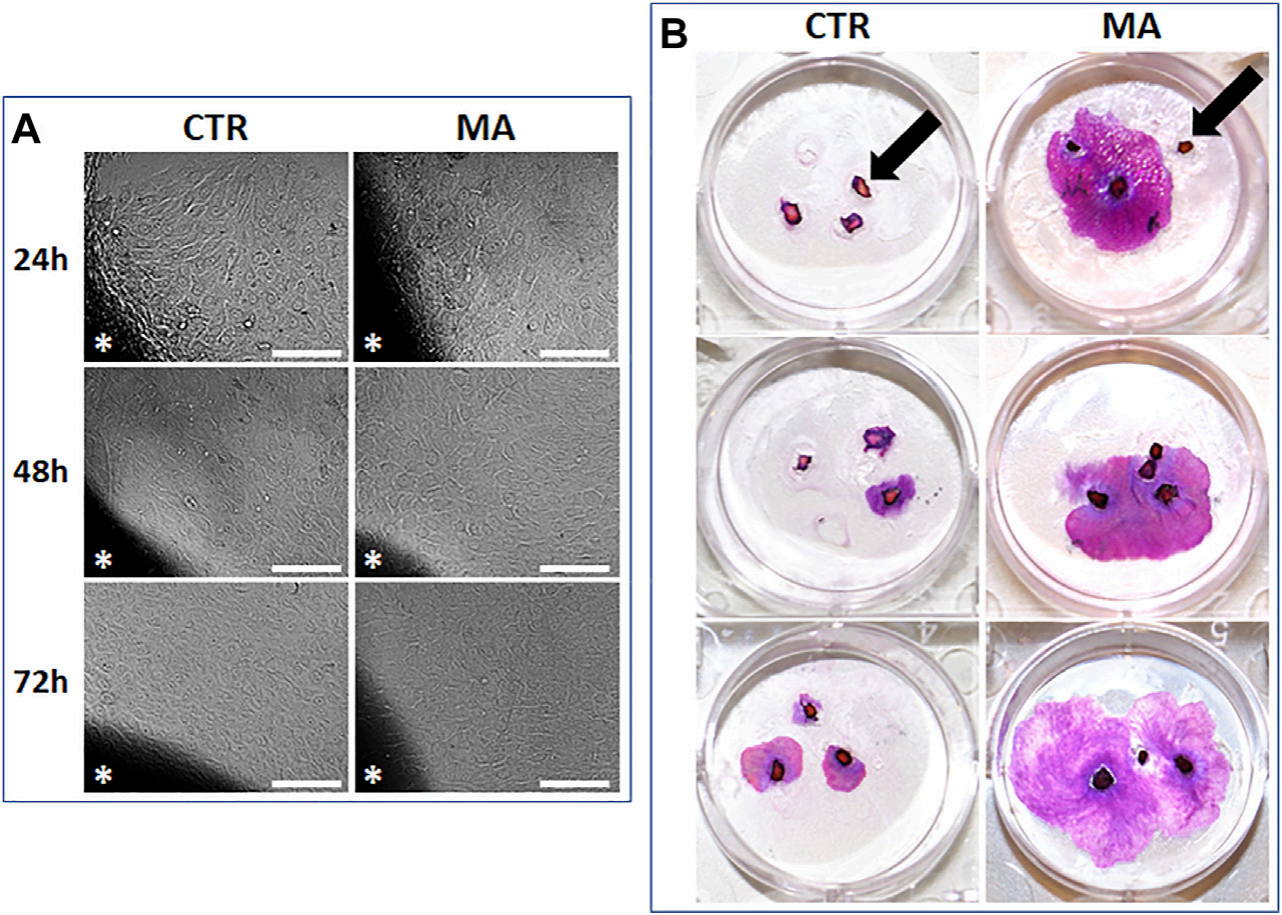
Generation of primary cultures of human skin keratinocytes using the explant technique (small tissue fragments were placed in direct contact with the culture surface to allow cells to migrate from the tissue to the culture flask). **(A)**: Phase contrast microscope images of primary keratinocytes migrating from the skin explants (labeled with asterisks) to the culture surface in each culture condition. Scale bars: 100 µm. **(B)**: Illustrative images of skin tissue explants cultured for 21 days in CTR and MA medium at a concentration of 5 μg/ml stained with hematoxylin-eosin. Some skin explants failing to generate any keratinocyte cultures are labeled with black arrows.

Once we determined that the concentration of 5 μg/ml of MA was able to improve cell proliferation of immortalized keratinocytes, we evaluated the effect of this concentration of MA on normal human skin keratinocytes. Results first showed that the percentage of explants able to successfully generate keratinocyte colonies significantly differed between CTR and MA groups at specific culture times. At day 7 of follow-up, we found that 50% of the explants cultured with CTR medium were able to efficiently generate keratinocyte colonies (Figure 4), whereas 58.33% of the explants cultured in MA showed keratinocyte colonies. Differences were not statistically significant at this time (*p* > 0.05). However, we found a significant increase of the culture efficiency after 14 days of culture, with 50.00% success rate in the CTR group and 66.67% in the MA group (*p* = 0.0214). Similarly, statistically significant differences (*p* = 0.0006) were found at day 21 (33.33% in CTR and 58.33% in MA).

Then, we quantified the surface area occupied by growing keratinocyte colonies after 21 days of culture using the explant technique and the cell dissociation method. Results of the explant technique (Figure 4) showed that 16.94 ± 13.39% of the culture surface was occupied by keratinocyte colonies in the CTR group, whereas 22.14 ± 16.09% corresponded to growing colonies in the MA group. Differences were statistically significant (*p* = 0.0185). In turn, quantification of the area corresponding to keratinocyte colonies in the group corresponding to enzymatic dissociation was significantly higher (*p* = 0.0315) in the MA group (1.7817 ± 2.4292%) than in the CTR group (0.2783 ± 0.6818%).

## 4 Discussion

The clinical management of severely burnt patients is challenging. In all cases, a rapid covering of the injured areas of the skin is critical to prevent water loss and infection, one of the main causes of death in these patients (Gacto-Sanchez, 2017). However, skin substitutes are highly dependent on the availability of primary cell cultures that typically require long periods of time and hamper the fabrication process of the bioartificial skin (Gardien et al., 2014). For this reason, optimization of the existing biofabrication protocols in skin tissue engineering is in need.

MA or (2α,3β)-2,3-dihydroxyolean-12-en-28-oic acid is a pentacyclic triterpene that is present in different plant extracts, including the olive oil (Lozano-Mena et al., 2014). As a natural molecule, MA demonstrated to be highly biocompatible and able to exert numerous important biological effects (Sánchez-Quesada et al., 2013; Lozano-Mena et al., 2014), what makes this compound an excellent candidate growth factor in tissue engineering. Since MA has not been previously used to improve the generation process of cultured human kin keratinocytes, we first demonstrated that MA was safe at the low concentration used and the cytotoxicity only appear at concentrations above 40 μg/ml, at least in the cells evaluated in the present work. The fact that cytotoxicity was only detected by one of the analytical methods is consistent with our previous reports showing that LIVE/DEAD methods are highly sensitive and able to detect early cell damage, whereas DNA quantification can only detect cell death when the cell membrane is severely disrupted at the last stages of the cell death process (Oliveira et al., 2014; Velasco-Ortega et al., 2016). Interestingly, the cytotoxic concentrations found in the present work are in agreement with previous reports demonstrating that the specific cytotoxic effects of MA are detectable at higher concentrations, with an IC50 of approximately 50 μg/ml in several cancer cell lines (Juan et al., 2008; Lozano-Mena et al., 2014). According to those previous works, the antitumoral activity of MA is mediated by an induction of cell cycle arrest at the highest concentrations of MA, showing non-specific cytotoxicity at concentrations above 100 μg/ml (Reyes et al., 2006; Lozano-Mena et al., 2014).

We have also shown that MA have a positive effect on cell proliferation at all tested concentrations. However, the only MA concentration showing significant pro-proliferative potential at the three time periods was 5 μg/ml. Although it is likely that several concentrations of MA could have potential usefulness in enhancing cell proliferation, selection of a concentration as low as 5 μg/mL has the additional advantage of being highly biocompatible and free from cytotoxic effects. Moreover, we have also demonstrated that this concentration of MA was capable of increasing the percentage of cells showing positive expression of the KI-67 protein as compared to control cells. This fact strongly supports the potential of MA to improve cell proliferation in culture keratinocytes. Although its functions are not completely understood, KI-67 is a bona-fide marker of cell proliferation that is commonly used to identify proliferating cells in a cell population (Juríková et al., 2016; Sun and Kaufman, 2018).

Therefore, the next step was to shed light on the putative mechanisms associated to the pro-proliferative activity of 5 μg/ml of MA. In this regard, we analyzed the mRNA expression of several genes playing a relevant role in epithelial cell function. This analysis confirmed the role of role of MA at 5 μg/ml in the upregulation of the *proliferating cell nuclear antigen* gene (*PCNA*), which is strongly related to cell proliferation, DNA replication and several replication-associated roles (Boehm et al., 2016). In this milieu, it is important to note that these gene expression analyses were carried out on non-confluent cells, since confluent cell cultures tend to show important differences with non-confluent cells, and cell density is a key factor modulating the gene expression pattern of several types of cells (Kim et al., 2014). Results also showed a positive effect on the expression of several genes related to epithelial cell differentiation and function: cytokeratins, intercellular junctions, epithelial differentiation and basement membrane. Although most of the assessed genes did not vary upon MA culture, the fact that some specific genes playing a role in epithelial differentiation are affected suggests that MA could contribute not only to enhance keratinocyte proliferation, but also to keep the orthotypical phenotype of these cells in culture. It is important to notice that maintaining the differentiated phenotype of cultured cells would contribute to the potential clinical use of these cells, since it is well known that most cultured human cells tend to dedifferentiate and lose their original properties in culture (Duan et al., 2017; Guo et al., 2017), and keratinocytes are not an exception to this rule (Hagedorn et al., 2009; Rosin et al., 2020). Although certain degree of dedifferentiation could be positive to favor cell proliferation, keratinocytes dedifferentiated in culture lose important physiological capabilities such as the potential to form microridges and express cytokeratins when clinically used (Hagedorn et al., 2009). These results are in agreement with previous reports suggesting that MA can induce cell differentiation of colon adenocarcinoma cells (Reyes et al., 2006) and can modulate cell function of cultured osteoclasts (Li et al., 2011). Future studies should be carried out on normal human keratinocytes to determine if the results found in immortalized cells can be reproduced in normal cells. In addition, the effect of MA on gene expression of other genes related to cell senescence, tumorigenesis and other undesired functions also needs to be furtherly determined.

Finally, to evaluate the activity of MA on normal human keratinocytes, we performed specific experiments on small skin biopsies taken from pediatric donors, resembling the clinical condition in which primary cultures of human keratinocytes are established. The results of these assays confirm previous results, demonstrating that MA is able to improve the efficiency of the explant, and that the percentage of successful explants was higher when 5 μg/ml of MA was used. Interestingly, this concentration of MA was associated to an improvement in the area of confluent keratinocyte colonies generated using both the explant and the cell dissociation protocols. Very few methods have been described to date to expand the efficiency of current protocols used in skin tissue engineering. Among these methods, we recently found that the use of nanoformulated EGF was also able to increase this efficiency applied to the human skin, although the effect was non-significant when the cell dissociation technique was used (Chato-Astrain et al., 2021). Nevertheless, it is important to note that none of these methods, i.e. MA and nanoformulated EGF, was able to dramatically increase the number of keratinocyte cells obtained in culture, although differences with the control were significant in both cases. In consequence, further methods with higher efficiency should be developed in the future, and the potential synergic effects of a combination of MA and nanoformulated EGF should be furtherly evaluated. It is well known that foreskin-derived human keratinocyte cultures may behave differently from other types of keratinocytes, and a recent transcriptomic analysis revealed significant differences in their metabolic profile, especially when foreskin cells are obtained from young children (Mangez et al., 2022). Further studies should be carried out to determine whether or not the results found in the present study can be confirmed in other types of normal human skin keratinocytes obtained from other sources and from adult donors. In addition, an unsolved question is the effect of MA on passaged normal keratinocyte cultures and if MA is capable of increasing the number of cell passages that can be performed on these cells. In this regard, it has been demonstrated that primary human cell cultures show a limited cell expansion potential, and the number of subcultures that can be performed on primary cultures is always limited (Martin-Piedra et al., 2014).

On the other hand, and even though our results support the hypothesis that cells expanded in the presence of MA could be potentially useful in tissue engineering, future studies should be carried out to determine the potential of these cells to generate bioartificial substitutes of the human skin able to fulfill the strict requirements of cells cultured for clinical use (Egea-Guerrero et al., 2019).

The present study has several limitations. First, the results from immortalized human keratinocytes need to be confirmed in other types of cells, since only one type of immortalized cells was used here. Second, alternative sources of normal human keratinocytes should be evaluated, including different skin origins and donor ages. Furthermore, the analysis of gene expression should also be performed on the normal cells in order to confirm the results obtained with the immortalized keratinocytes.

In summary, the present work demonstrated that MA could exert a positive influence on the efficiency of current tissue engineering protocols applied to the biofabrication of a biological substitute of the human skin. The high biocompatibility of this component (Lozano-Mena et al., 2014), along with its availability and affordability as a natural product support its use in skin tissue engineering and opens the door to other tissue engineering applications in cornea, oral mucosa and other human tissues and organs.

## Data Availability

The original contributions presented in the study are included in the article/Supplementary Material, further inquiries can be directed to the corresponding authors.
