# Supplementary material for: Biological Effects of Maslinic Acid on Human Epithelial Cells Used in Tissue Engineering
Source: Front Bioeng Biotechnol. 2022 Apr 27;10:876734. doi: 10.3389/fbioe.2022.876734 (PMC9159156; doi:10.3389/fbioe.2022.876734)
Supplement: Supplementary file 1 [file Table1.docx]

**Supplementary Table S1**. Cell proliferation of immortalized human keratinocytes as determined by sequential cell number quantification and WST-1 method. Cells were cultured for 24h, 48h and 72h in control basal media (CTR) and media containing increasing concentrations of maslinic acid (MA). For the cell proliferation, both the absolute number of cells and the percentage of cells (using the CTR as a 100% reference) are shown. For WST-1, values correspond to the percentage of enzymatic activity using the CTR as a 100% reference. Statistical p values correspond to the comparison of each value with those obtained for the CTR group using Mann-Whitney tests. Statistically significant values are highlighted with asterisks (*).

| **TIME** | **MA CONCENTRATION** | **CELL COUNT (NUMBER OF CELLS)** | **PERCENTAGE OF CELLS (%)** | **CELL P VALUE** | **WST-1 (%)** | **WST-1 P VALUE** |
| --- | --- | --- | --- | --- | --- | --- |
| **24 h** | **CTR** | 3155±1130.15 | 100±38.19 | - | 100±16.58 | - |
|  | **1 µg/mL** | 4507.5±1080.61 | 145.71±36.52 | 0.0542 | 108.36±3.81 | 0.0547 |
|  | **5 µg/mL** | 5278.33±1074.61 | 171.75±36.31 | 0.0250* | 147.3±6.52 | 0.0039* |
|  | **10 µg/mL** | 5129.17±464.58 | 166.71±15.7 | 0.0039* | 128.72±3.89 | 0.0547 |
|  | **20 µg/mL** | 5495.83±1461.69 | 179.1±49.4 | 0.0250* | 150.55±4.26 | 0.0039* |
|  | **40 µg/mL** | 5268.33±630.04 | 171.42±21.29 | 0.0039* | 139.08±6.96 | 0.0104* |
|  | **80 µg/mL** | 5380.83±566.49 | 175.22±19.14 | 0.0039* | 157.08±3 | 0.0039* |
| **48 h** | **CTR** | 3754.17±1156.84 | 100±33.75 | - | 100±11.41 | - |
|  | **1 µg/mL** | 5282.5±851.72 | 144.59±24.85 | 0.0250* | 114.97±10.59 | 0.0374* |
|  | **5 µg/mL** | 4962.5±769.39 | 142.77±14.37 | 0.0176* | 126.96±9.09 | 0.0039* |
|  | **10 µg/mL** | 5605.83±741.74 | 154.02±21.64 | 0.0104* | 138.65±9.25 | 0.0039* |
|  | **20 µg/mL** | 5115.83±338.49 | 139.73±9.88 | 0.0065* | 157.34±7.91 | 0.0039* |
|  | **40 µg/mL** | 6595.83±1629.1 | 182.91±47.53 | 0.0250* | 157.3±12.12 | 0.0039* |
|  | **80 µg/mL** | 3011.67±312.5 | 78.34±9.12 | 0.1495 | 195.5±1.18 | 0.0039* |
| **72 h** | **CTR** | 4556.67±1316.17 | 100±29.99 | - | 100±13.81 | - |
|  | **1 µg/mL** | 4290.83±1197.64 | 93.94±27.29 | 0.7488 | 131.04±19.52 | 0.0285* |
|  | **5 µg/mL** | 7102.5±1397.96 | 158.01±31.86 | 0.0163* | 126.37±6.32 | 0.0062* |
|  | **10 µg/mL** | 4477.5±1595.95 | 98.2±36.37 | 0.8728 | 157.34±14.15 | 0.0062* |
|  | **20 µg/mL** | 5575±390.35 | 123.21±8.9 | 0.1093 | 188.14±13.28 | 0.0062* |
|  | **40 µg/mL** | 4196.67±1867.63 | 91.8±42.56 | 0.5218 | 193.25±22.94 | 0.0062* |
|  | **80 µg/mL** | 1265.83±251.7 | 25.01±5.74 | 0.0039* | 177.09±11.71 | 0.0062* |
