# Supplementary figures and images for: Biological Effects of Maslinic Acid on Human Epithelial Cells Used in Tissue Engineering
Source: Front Bioeng Biotechnol. 2022 Apr 27;10:876734. doi: 10.3389/fbioe.2022.876734 (PMC9159156; doi:10.3389/fbioe.2022.876734)

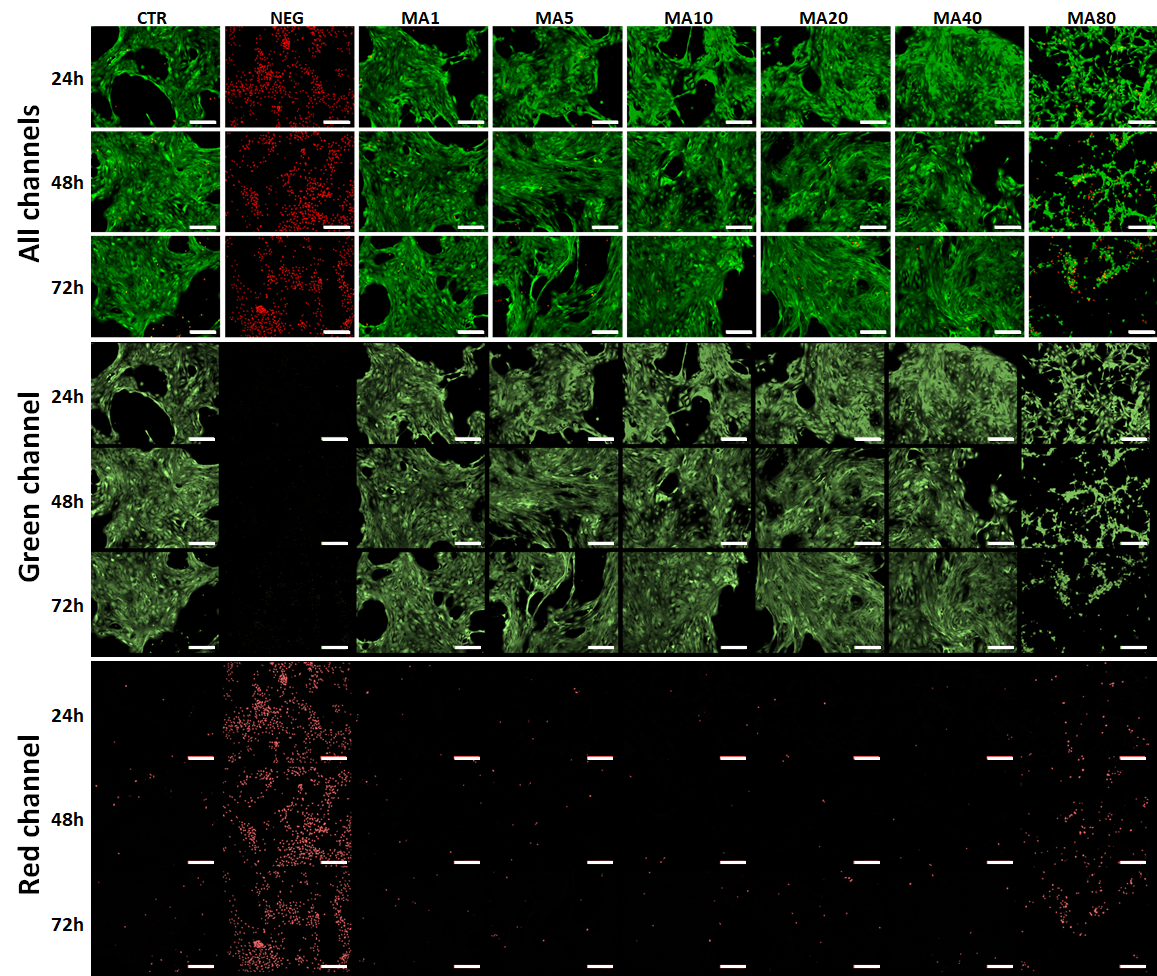

Supplement: Supplementary file 2 [file Image1.TIF]
